# Supplementary material for: Analysis of chi angle distributions in free amino acids via multiplet fitting of proton scalar couplings
Source: Magn Reson (Gott). 2024 Aug 19;5(2):103–20. doi: 10.5194/mr-5-103-2024 (PMC11570886; doi:10.5194/mr-5-103-2024)
Supplement: The supplement related to this article is available online at: https://doi.org/10.5194/mr-5-103-2024-supplement. [file mr-5-103-2024-supplement.zip › Syed2024/data/fit1d_fitnmr_output.tar.gz!/fit1d/ser/500/bmse000048_simulation_1_nmredata/1H/resonances_1d.pdf]

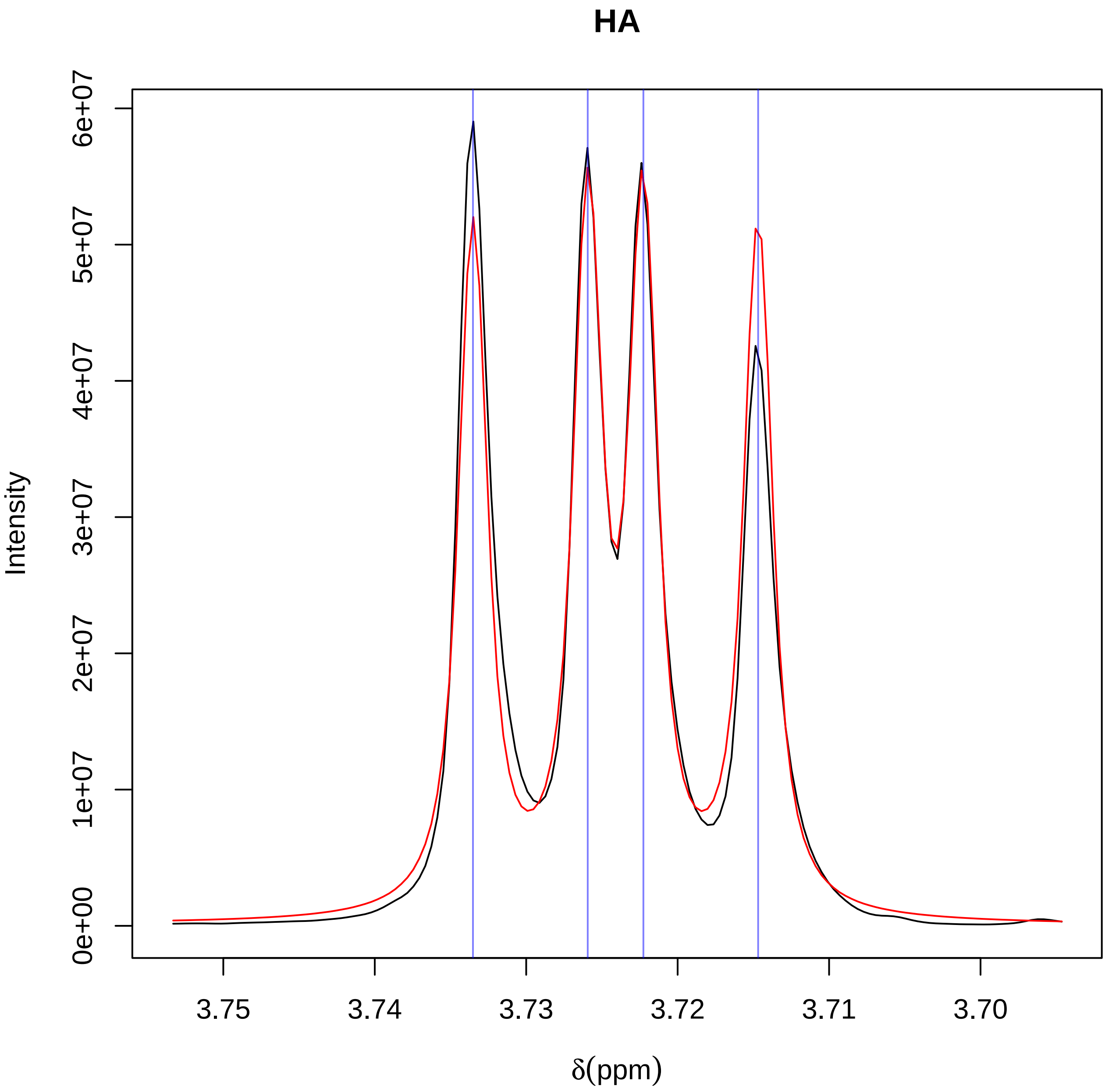

HA
Intensity
0e+00
1e+07
2e+07
3e+07
4e+07
5e+07
6e+07
3.75
3.74
3.73
3.72
3.71
3.70
δ(ppm)

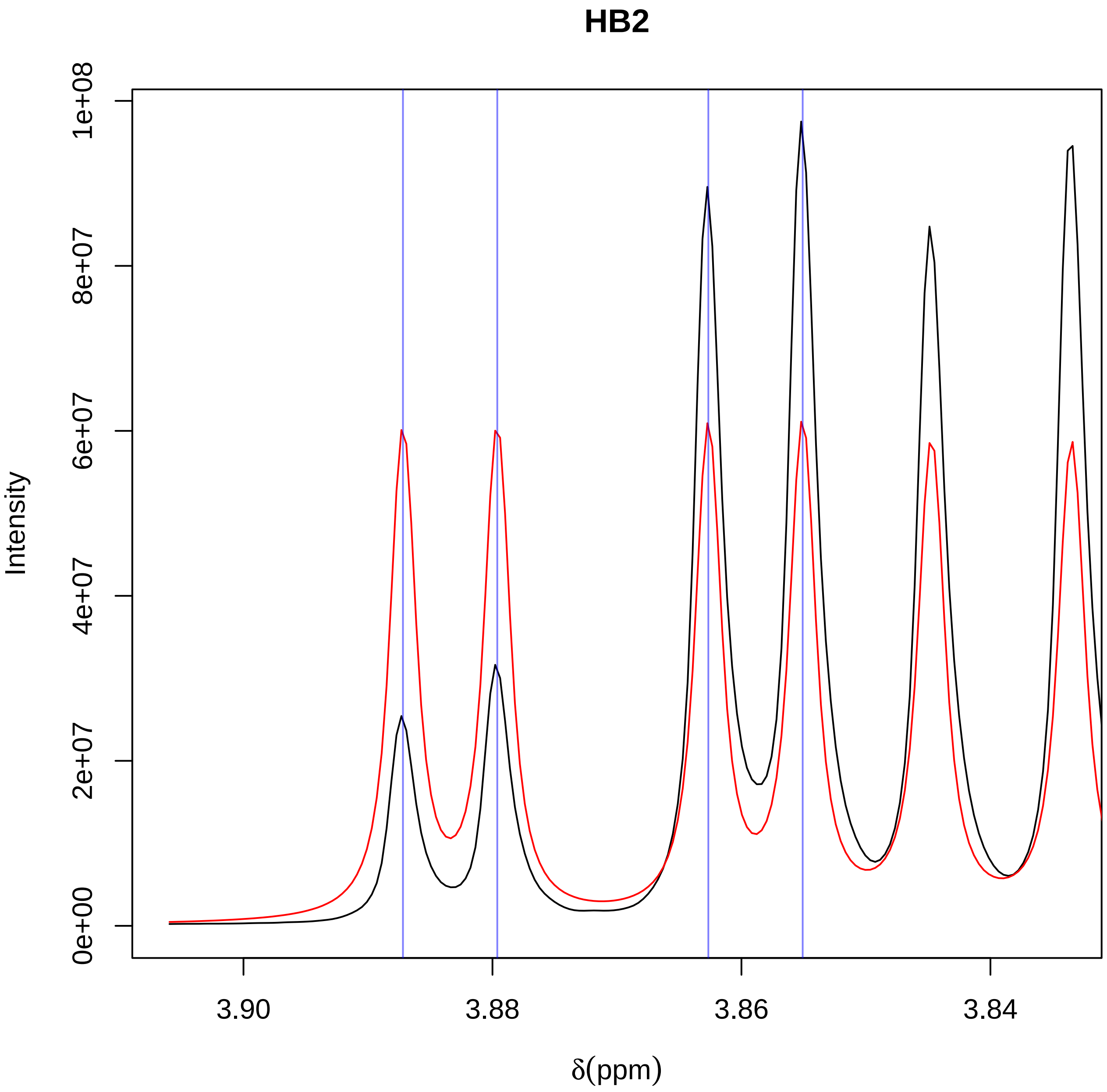
HB2
Intensity
0e+00
2e+07
4e+07
6e+07
8e+07
1e+08
3.90
3.88
3.86
3.84
δ(ppm)

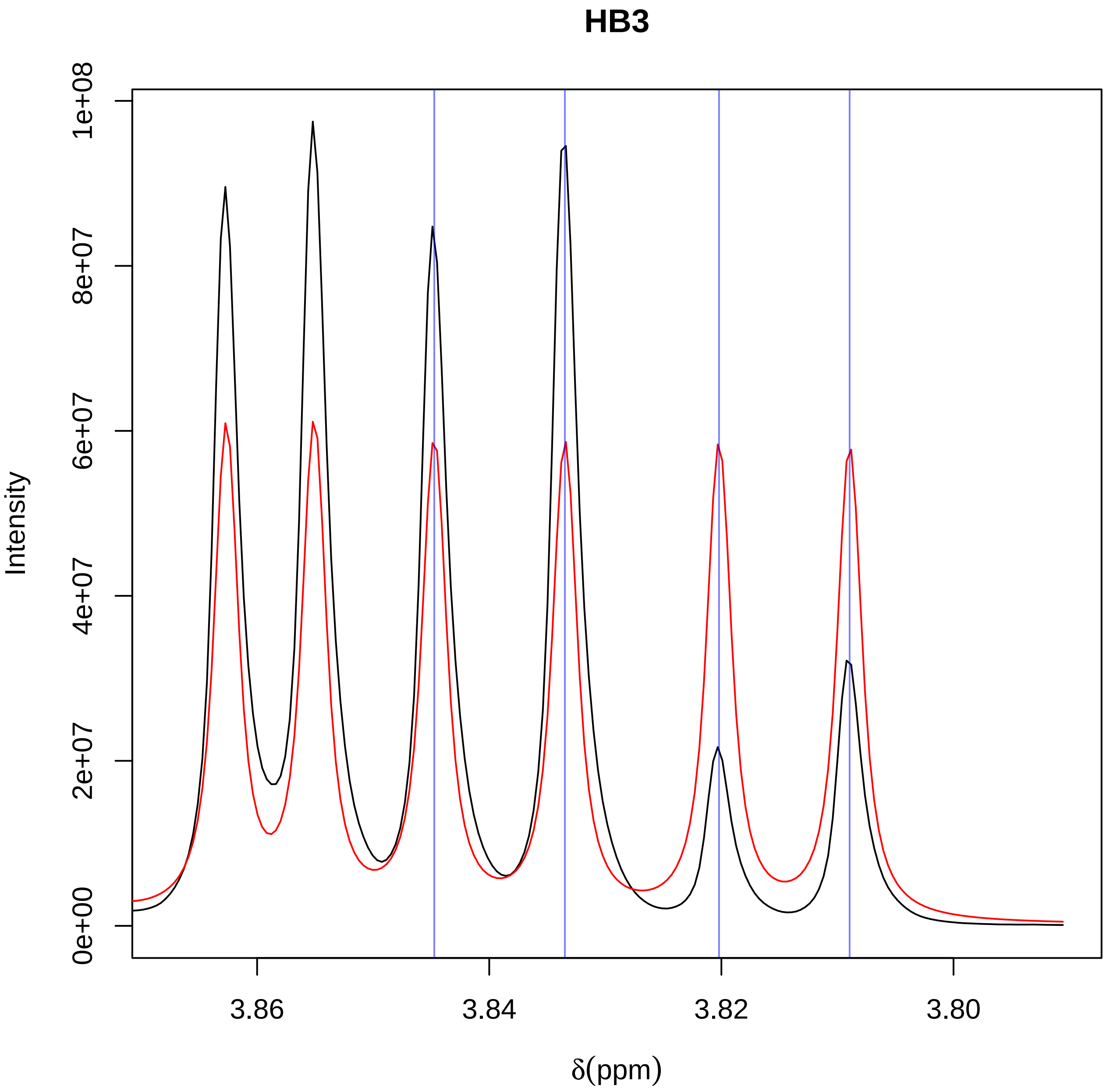

HB3
Intensity
0e+00
2e+07
4e+07
6e+07
8e+07
1e+08
3.86
3.84
3.82
3.80
δ(ppm)
